# Could the organ shortage ever be met?

**DOI:** 10.1186/s40504-015-0023-1

**Published:** 2015-07-23

**Authors:** Mairi Levitt

**Affiliations:** Department of Politics Philosophy & Religion, County South College, Lancaster University, Lancaster, LA1 4YD UK

**Keywords:** Organ donation, Organ shortage, Organ transplants, Presumed consent, Northern Ireland

## Abstract

The organ shortage is commonly presented as having a clear solution, increase the number of organs donated and the problem will be solved. In the light of the Northern Ireland Assembly’s consultation on moving to an opt-out organ donor register this article focusses on the social factors and complexities which impact strongly on both the supply of, and demand for, transplantable organs. Judging by the experience of other countries presumed consent systems may or may not increase donations but have not met demand. Donation rates have risen considerably in all parts of the UK recently but there is also an increasing demand for organs. Looking at international donation rates and attitudes, future demand for organs and education on donation, the question is whether the organ shortage could ever be met. The increase in longevity, in rates of diabetes and obesity and in alcohol related liver disease all contribute both to increased demand for transplants, and re-transplants, and a reduction in the number of usable organs. It is unlikely that demand could ever be met, since, if supply was unlimited, the focus would move to financial resources and competing demands on the health care budget in a publicly funded health system. These factors point to the need to focus on ways of reducing, or at least stabilizing, demand where lifestyle factors contribute to the underlying disease.

## Supply and demand are rising

‘Boosting organ donation rates is the key to reducing the number of people who die waiting for a transplant’ (Northern Ireland Executive 2013).

Edwin Poots, the Minister for Health, speaking at a meeting organised by the BMA Northern Ireland in February 2013, expressed a commonly held view on solving the organ shortage; increase the supply. It may seem appropriate to say that solving the organ shortage is a straightforward health care issue that can be tackled by all if us, as potential donors and next of kin, ensuring that more organs are donated. In this view, since the overwhelming majority of the British public are in favour of organ donation, to improve donor rates we could work on educating the public to increase the numbers with donor cards and to encourage discussion within families, and, given the high percentage in favour of donation, we should consider an opt-out system of presumed consent. Those few who are opposed would be able to make their wishes clear and the remaining majority will be passively enrolled.

In the UK as a whole there has been a 50 % increase in organ donation in five years, meeting the UK Organ donation taskforce target, with a further rise in 2013–14 (Table 1).

**Table 1:** Deceased organ donors, UK, 2012/13 compared with 2007/08 by nation of donor hospital and 2013/14 figures

	2007/08	2012/13	% increase	2013/14
England	688	1026	49.1	1097
Wales	45	52	15.6	60
Scotland	54	94	74.1	108
N Ireland	22	40	81.8	48
Total	809	1212	49.8	1313

Please note that % increase for Wales for 2011/12 over the 2007/8 baseline was 49 % (they had 67 deceased donors in that year)*

Source: NHS Blood and Transplant News Release 11^th^ April 2013 http://www.nhsbt.nhs.uk/news-and-media/news-articles/news_2013_04_11.asp and NHSBT Overview of Organ donation and transplantation 2013–14 figure 2.1. p.7 http://nhsbtmediaservices.blob.core.windows.net/organ-donation-assets/pdfs/overview.pdf

*In Wales the op-out system is due to be introduced in 2015 but no specific evidence has either linked the fall in donor numbers between 2011/12 and 2012/13 to the proposed opt out legislation or ruled out a link

### Infrastructure rather than attitudes

If there had been a change to a presumed consent system we might have thought that was the reason for the increase in donation but the key seems to be the infrastructure now in place. 100 donor coordinators are working in 18 teams and 12 in-house co-coordinators based full time in single critical care units or Trusts (BMA Medical Ethics Committee 2012). The number of specialist nurses has more than doubled since the 2008 Task Force report, to 250. These nurses offer continuing support to the donor family, staying with them throughout the process. As might be expected consent rates are higher when a specialist nurse is there for the families (68 %: 37 % in 2012/13) (NHS Blood and Transplant 2013 p.125). There are also higher rates of referral from the emergency department and a network of organ donation champions in hospitals to increase awareness among medical staff, especially in ICU and emergency care.

An article in Nursing Standard reports that consent rates in families approached by specialist nurses are high and that ‘it is thought that the extra training in communication skills may be responsible’ (Mercer 2013 p.38). The USA donation rates increased not by a focus on education and attitudes but with a focus on providing an integrated donation process with early referral and the ‘aggressive pursuit of every donation opportunity’ (Grazier 2011 p.198). Evidence that infrastructure is crucial comes from Spain where the rise in donation rates came years after the 1979 presumed consent legislation. In 1989, ten years after the legislation, there were still only 14.3 donors per million population donors but this had increased to 35.1 by 2005 (Navarro-Michel 2011 p.151). Attitudes seem less important since, according to EU research, exactly the same percentage of respondents in Spain and UK reported that they were willing to donate an organ immediately after their death (61 %) and more UK respondents were willing to donate the organs of a deceased close family member if asked (64 % UK: 59 % Spain) (Eurobarometer 2010). In practice UK families have been far more likely to refuse consent when a decision had to be made and here the specialist staff, influenced by the Spanish model, appears to make a difference. Interestingly, the UK has a far higher rate of living organ donors which brings the rate for organ donations as a whole closer to the Spanish level (Rudge et al. 2012).

The expectation is that a change to presumed consent will increase the number of donors. While the raw figures do not show a significant difference between countries with different consent models, a study that controlled for ‘other determinants of organ donation’, including religious belief and number of deaths in traffic accidents, found cadaveric donation rates to be 25 to 30 % higher in presumed consent countries while rates of living donation tend to be lower (Bell and Harris 2010; Abadie and Gay 2004).

### Presumed consent and waiting lists

Spain is taken to be the gold standard for deceased organ donation and may be close to the limit in terms of the rate of deceased organ donors that can be achieved (Navarro-Michel 2011 p.155). Despite this Spain has around 5500 on the organ transplant list which, given the smaller population, is a similar proportion to the UK’s figure of 7000. Presumed consent has therefore not met the demand for transplants in Spain which has a soft opt out model in which families are still consulted. Singapore adopted a hard presumed consent system in 1987 in which organs are removed unless the deceased has opted out prior to death (Ministry of Health Singapore 2005). Muslims in Singapore were opted out as a group for 20 years but were suffering from a shortage of organs and have been included since 2008. Despite this system the waiting list was reported to be 500 in a population of 5 million (The Strait Times 2013).

A report from Singapore of relatives begging doctors to give a patient a little longer time in case he recovered before retrieving the organs generated newspaper headlines supporting the law; for example, ‘Postponement killed dreams of liver transplant patients’. The family had been granted a 24 hour delay and the article quoted the health minister reporting in parliament that the delay rendered the liver unusable but the corneas and kidneys were retrieved (Berger 2007). It seems likely that media reaction in the UK would be more mixed.

Under the current system there is often a degree of doubt over the patient’s wishes. If patients have not registered their wishes or talked about donation the family may still consent, on the grounds, for example, that s/he was the sort of person who wanted to help others. However, under presumed consent if someone has registered to opt out then the family could not be approached.

It might be argued that an opt out system is out of step with the Human Tissue authority (HTA) guidance on consent which covers England, Wales and Northern Ireland. ‘For consent to be valid it must be given voluntarily, by an appropriately informed person who has the capacity to agree to the activity in question’ (Human Tissue Authority 2009). Joining the organ donation register is not consent in the way that is understood in medicine. The consent form has a tick box ‘I want to donate the following for transplantation after my death’ (NHS Blood and Transplant). Children can join with parental permission – and around 30 % join age 16 to 25. There is no process for re-consent. In the HTA code of practice it is stated that;

‘It is important to establish clearly when consent has been given, to ensure the removal, storage or use of any tissue is lawful. However, giving consent should not be seen as a single act – the signing of a consent form. Rather, it should be seen as part of a continuing process in which individuals, and their relatives or close friends, can discuss the issue fully, ask questions and make an informed choice’ (NHS Blood and Transplant para.68).

On the other hand, organ shortage may be characterised as a uniquely important problem in healthcare where the end justifies the means or it might be argued that presumed consent or even compulsion should be used in other areas. What is clear is that demand is rising and has not yet been met by increased donations, even with the hard consent model used in Singapore.

### Rising demand

The numbers suffering from diseases that may lead to the need for a transplant is rising, particularly the incidence of type 2 diabetes and liver disease both of which are linked to lifestyle factors. The number of people diagnosed with diabetes in the UK increased by nearly 130,000 to 2.9 million in 2011; this is an almost 50 % rise since GPs first published diabetes data in 2005 (Diabetes UK 2012). The rise is mainly in Type 2 diabetes, which accounts for around 90 % of all diagnoses. One third of those with diabetes will develop a kidney disorder. Not all the risk factors for type 2 diabetes can be controlled, notably age, family history and ethnic group, but lifestyle factors are also important. According to Diabetes UK 80–85 % of the overall risk of type 2 diabetes is from being obese or overweight.

The incidence of alcoholic induced liver disease is also rising. In England the numbers treated in hospital for alcohol related liver damage has risen by 1000 a year from 2009–12 and includes younger drinkers under the age of 30. The number of livers donated to people with alcohol-related liver disease has also risen from 146 in 2009 to 197 in 2012 which was over a quarter of the total number of the liver transplants performed in the UK (Mail on-line 2013).

The characteristics of donors in the UK mean that the transplant rates have not improved as much as the donation rates. Donors are older, more obese, and less likely to have suffered a trauma-related death, all of which reduce the number of usable organs. In 2013–14 37 % of deceased donors were over the age of 60 and 24 % were clinically obese (NHS Blood and Transplantation 2014 p.17). More donations are made after cardiac death and, on average, fewer organs are retrieved than from donations after brain death. Finally the success of transplants increases the demand for re-transplants, where this is possible. According to the National Kidney Federation (2014) only half the kidneys transplanted from deceased donors last ten years or more. Around 20 % of kidney transplants are re-transplants.

For these reasons it may never be possible to meet the demand for organs but there is some scope for reducing demand. The BMA Medical Ethics Committee 2012 makes one reference to reducing demand in its report on organ donation;

‘…another equally important and complementary way of reducing the number of unnecessary deaths is to focus on public health measures to reduce the incidence of chronic diseases that lead to the need for an organ transplant’ (BMA op cit p.3).

The organ donation task force made a recommendation in the supplementary report, the only recommendation in the form of a question; Should we engage the public with the discourse of ‘disease prevention’ as well as organ donation? (Department of Health 2008 p.18). The reason for doubt cannot be that there is no scope for ‘disease prevention’ but perhaps that the straightforward message of giving life will be complicated and the public might see some recipients as less worthy of such a gift (Levitt et al. 2011). 

### Unlimited organs would raise resource issues

It is now recognised that building new roads to reduce queues does not have the desired effect for long. With shorter journey times more people travel and the new roads are soon clogged up again (Department of Transport, 1994; Goodwin 1996, Næss et al. 2012). Road building has not stopped, but at the same time there is a need to think of ways to reduce demand. In a similar way, increasing the organ supply and maintaining it at higher levels will not solve the shortage because the number of organs required is not static. Demand is increasing and as organ supply increases then more people could be allowed on to the waiting list. If there was an unlimited organ supply, for example through xenotransplantation or tissue engineering, then attention would switch from supply to the resourcing of transplants and re-transplants and whether these should be offered to any patient whose life would be prolonged or death delayed. Presumed consent may or may not increase organ donations in any particular country but even with the improvements in infrastructure it is not enough to meet the rising demand (Hitchen 2007). This is not say that organ supply should be neglected but that demand and supply should be tackled together. Organ donation will prolong the lives of recipients but increased donations will not on their own solve the organ shortage. If demand is to be stabilised then education needs to focus on preventable health conditions that lead both to organ failure and to a reduction in the usability of organs that are donated.
